# Gaming Disorder in Adults with Autism Spectrum Disorder

**DOI:** 10.1007/s10803-021-05138-x

**Published:** 2021-06-28

**Authors:** Alayna Murray, Arlene Mannion, June L. Chen, Geraldine Leader

**Affiliations:** 1 Irish Centre for Autism and Neurodevelopmental Research (ICAN), School of Psychology, National University of Ireland, Galway, Ireland; 2 Department of Special Education, Faculty of Education, East China Normal University, Shanghai, China

**Keywords:** Autism spectrum disorder, Addiction, Gaming disorder, Video game, Internet, Gelotophobia

## Abstract

Gaming disorder (GD) is a clinical addiction to video or internet games. This study investigated whether GD symptoms are heightened in adults with autism spectrum disorder (ASD) in comparison to a control group, and explored predictors of GD in 230 adults with ASD and 272 controls. The relationship between GD and gelotophobia was examined. Measures included the Ten-Item Internet Gaming Disorder Test, GELOPH < 15 >, Autism Spectrum Quotient-10 items, Inventory of Parent and Peer attachment, Emotional Regulation Questionnaire, Social Functioning Questionnaire (SFQ) and the NEO-FFI-3. Individuals in the ASD group showed significantly higher symptoms of GD. Peer-attachment, emotional regulation and extraversion significantly predicted GD scores. Gelotophobia and GD were related to each other with a small effect size.

## Introduction

Autism spectrum disorder (ASD) is characterised by persistent impairments in social interaction and communication in addition to restrictive, fixated and repetitive patterns of thought, behavior and interests (American Psychiatric Association, 2013). The American Center for Disease Control and Prevention (CDC) evaluated prevalence rates for ASD and found that approximately one in 54 children were diagnosed with ASD (Maenner et al., 2020) Autism spectrum disorder symptoms present on a wide spectrum and ASD can be diagnosed at three levels. Level 1 diagnoses indicate that an individual requires support, level 2 diagnoses imply that an individual requires substantial support, and level 3 diagnoses are characterised by individuals who require very substantial support (American Psychiatric Association, 2013).

Individuals with ASD are more likely than TD individuals to be diagnosed with other medical conditions such as behavioral problems, feeding problems, toileting issues, gastrointestinal symptoms, epilepsy, sleep problems and attention-deficit/hyperactivity disorder (Devlin et al., 2008; Leader & Mannion, 2016a, 2016b; Leader et al., 2020, 2018b; Mannion & Leader, 2013, 2014a, 2014b, 2016 ) as well as numerous psychiatric conditions. Recently, a meta-analysis was conducted to evaluate ASD and psychiatric conditions and it was found that 54.8% of individuals with ASD presented with a psychiatric disorder of some form (Lugo-Marín et al., 2019).

Addiction has traditionally been related to psychoactive substances (e.g. alcohol, heroin) where there is physical dependence on the substance (Chamberlain et al., 2016). However, in recent years, experts have debated that at excessive levels, many behaviors can be considered compulsive and addictive. These behaviors include gambling, shopping, internet use, videogame play and sexual behaviors (Leeman & Potenza, 2013). Gaming disorder (GD), an addiction to games, is a behavioral addiction which can be more prevalent in individuals with ASD compared to TD individuals (Murray et al., 2021). GD was listed in the DSM-5 under the emerging measures section where it needs further research. Under the proposed criteria, there are nine symptoms of GD, five of which must be present over a 12-month period to warrant a diagnosis: preoccupation with videogames, withdrawal symptoms, tolerance build-up, a need to game at increasing levels to feel satisfied, unsuccessful attempts to quit or control gaming, loss of interest in hobbies as a result of gaming, continued intensive use of internet games despite problems, deceiving others regarding the amount of time spent gaming, use of games to escape negative moods and jeopardising or losing a job, relationship or other important opportunity because of gaming (American Psychiatric Association, 2013).

Since the release of the DSM-5 (American Psychiatric Association, 2013), the World Health Organization has defined gaming disorder as a clinical behavioral addiction in the International Classification of Disease, Eleventh Revision (ICD-11) which will come into effect in 2022 (World Health Organization, 2018). The criteria for a diagnosis overlap with the DSM-5 and must be present for 12 months: reduced control over gaming, increase in priority given to games to the extent that it negatively effects hobbies and the escalation and continuation of gaming regardless of negative consequences in one’s personal life (World Health Organization, 2018).

It is hypothesised that individuals with ASD may be more susceptible to developing GD, both due to the fact that gaming can be a restricted interest (Mazurek & Engelhardt, 2013), and due to internet gaming being a safer space where an individual with ASD may feel less social pressure (Benford & Standen, 2009). Research investigating GD in adults with ASD is sparse (Craig et al., 2021; Murray et al., 2021), with most studies examining samples of children and adolescents (e.g. Mazurek & Engelhardt, 2013; Mazurek & Wenstrup, 2013). The research in young samples has found that individuals with ASD show higher rates of GD symptomology in comparison to typically developing (TD) controls with medium and large effect sizes (MacMullin et al., 2016; Mazurek & Engelhardt, 2013; Mazurek & Wenstrup, 2013; Paulus et al., 2019). Only one study has investigated the relationship between ASD and GD in an adult sample, which found similar results to the studies above looking at youth with ASD. The study revealed a strong significant relationship between ASD and GD when controlling for daily gaming hours and proportion of free time spent gaming (Engelhardt et al., 2017). There are some gaps in the literature on GD and ASD; in these studies, no participant was over the age of 25, there were significantly more males than females, and no studies have investigated correlators or predictors of GD in samples with ASD other than daily gaming hours and proportion of free time spend gaming.

Gelotophobia is defined as a fear of being laughed at, made fun of or ridiculed (Titze, 2009). “Gelos” is the Greek word for laughter (Ruch & Proyer, 2008a) and “phobia” means fear. Gelotophobia can be mild, moderate or severe, although severe gelotophobia is somewhat rare, particularly in TD samples (Ruch & Proyer, 2008b). When a person is mocked, they can experience negative feelings such as embarrassment, shame, anger and disgust (Platt, 2008). However, most people adapt quickly and modify their behavior to act accordingly to the situation (Tsai et al., 2018). There is a subgroup of people who do not adapt to these situations and believe that others mock them due to their own social inadequacy. Gelotophobes believe something is entirely wrong with them and that they are “intolerably ridiculous” (Titze, 2009, p. 30). Gelotophobia is particularly prevalent in individuals with social anxiety (Edwards et al., 2010), those with deficits in understanding and describing emotions (Boda-Ujlaky & Séra, 2013), and in those who don’t understand or appreciate humour as well as others (Chan, 2016; Hiranandani & Yue, 2014). Furthermore, these factors themselves are strongly linked with ASD, which is indicative that those with ASD may be predisposed to gelotophobia. Indeed, previous research has demonstrated significantly higher rates of gelotophobia in participants with a diagnosis of ASD (Grennan et al., 2018; Leader & Mannion, 2020; Leader et al., 2018a).

No studies to date have investigated the link between gelotophobia and GD. However, gelotophobia and GD are both particularly prevalent in individuals with social anxiety (Edwards et al., 2010; Sioni, et al., 2017), individuals with deficits in understanding and describing emotions (Boda-Ujlaky & Séra, 2013; Bonnaire & Baptista, 2019), individuals with lower self-esteem (Hiranandani & Yue, 2014; Van Rooij et al., 2014) and individuals with insecure attachment styles (Miczo, 2017; Schimmenti, et al., 2014). Hence, it is plausible that GD and gelotophobia themselves may be related.

The first aim of the present study was to examine the relationship between ASD and GD, the hypothesis is that adults with ASD will present with increased gaming disorder symptoms in comparison to the TD control group. The second aim was to investigate the predictors of GD in participants with and without ASD, including social functioning, extraversion, emotional regulation, and peer attachment. It is hypothesised that these variables will predict GD symptoms in adult participants with and without ASD. The final aim of the study was to evaluate if GD and gelotophobia are related to each other. It is hypothesised that individuals who demonstrate heightened symptoms of gelotophobia will also show more symptoms of GD.

## Method

### Participants

Participants were 230 adults with a diagnosis of ASD and were recruited through social media, support groups and organizations for individuals with ASD. Diagnoses were provided by psychiatrist or licensed psychologist independent of this study, as indicated by participants. Information on the professional(s) who made the diagnosis was obtained. The control group included 272 adults recruited from social media, through posters, and from a research participant system for university students in exchange for course credit. The mean age of the ASD group was 31.32 years (*SD* = 11.03) and the mean age of the control group was 29.51 years (*SD* = 13.53).

Participants (*n* = 23) were removed from the control group if they scored over the cut-off for ASD as set out by the Autism Spectrum Quotient (AQ-10) or if they were in the process of being diagnosed. Participants in the ASD group were required to disclose if they had a formal diagnosis and participants with self-reported diagnoses were excluded from the study (*n* = 2). No additional information was obtained to verify ASD diagnoses.

### Procedure

Adults over 18 years of age were recruited to participate in the present study. Participants were made aware of the study on social media, from posters, through a research system for psychology students in exchange for course credits, and through autism support and research groups. If an individual was interested in participating, they were provided with a participant information sheet and consent form. Once consent was obtained, informants could fill out the battery of measures below.

### Measures

#### Ten-Item Internet Gaming Disorder Test

The Ten-Item Internet Gaming Disorder Test (IGDT-10; Király et al., 2017) is a screening item to evaluate GD symptomology based on DSM-5 criteria. Questions evaluate the nine DSM-5 criteria for GD. Answers are displayed on a three-point scale (never = 0, sometimes = 0, often = 1) with a maximum score of nine. Although not diagnostic, a score of five or over was considered the cut-off point for GD for the purpose of this research. Recently, a cross-cultural validation study reported internal validity as reliable in an English-speaking sample, with a Cronbach alpha coefficient of 0.77 (Király et al., 2019). The alpha score in the present study was 0.75.

#### *GELOPH* < *15* >

The Geloph < 15 > (Ruch & Proyer, 2008b) is a 15-item subjective measure of gelotophobia symptomology. Cut-off scores can indicate if a person has no gelotophobia, slight, marked or severe gelotophobia. The alpha score in the present study was 0.93.

#### Emotional Regulation Questionnaire

The Emotional Regulation Questionnaire (ERQ; Gross & John, 2003) is a 10-item measure of emotional regulation. Emotional regulation is the capacity to control and regulate one’s emotions and the way a person deals with emotions. The scale includes two subdomains: cognitive reappraisal, and expressive suppression. The capacity of a person to consciously change their thoughts, beliefs and feelings when presented with an emotional event is known as cognitive reappraisal. Expressive suppression is a measure of a maladaptive coping strategy where a person suppresses their emotions. Alpha scores for both cognitive reappraisal (0.87) and expressive suppression (0.77) were good in the present study.

#### Autism Spectrum Quotient 10-items

The Autism Spectrum Quotient 10-items (AQ-10; Allison, et al., 2012) is a questionnaire which measures autistic traits in adults in the normal intelligence range. The AQ-10 includes items for each diagnostic symptom of ASD: social interaction, communication, attention to detail, attention switching and imagination (Booth et al., 2013). The Cronbach alpha coefficient for the present study was good (0.83).

#### Social Functioning Questionnaire

The Social Functioning Questionnaire (SFQ; Tyrer et al., 2005) is an 8-item measure of social functioning in the areas work and home tasks, familial relationships, financial concerns, sexual activity, personal relationships, and spare time activities. Answers are presented on a 4-point scale. The SFQ ranges from 0 to 24, with lower scores demonstrating better functioning. The Cronbach alpha coefficient in this study was 0.68.

#### Inventory of Parent and Peer Attachment

The Inventory of Parent and Peer Attachment (IPPA; Armsden & Greenberg, 1987) subjective measure of attachment to parents or peers. The peer-attachment facet (25-item) of this measure was implemented in this study to examine peer-attachment in participants. The 25-item measure is answered on a 5-point scale. Peer attachment is measured in three areas, trust, communication, and alienation. communication, trust, and alienation. Cronbach alpha scores were good in the current study ranging from 0.74 to 0.95.

#### NEO Five-Factor Inventory-3 (Extraversion Facet)

The NEO-FFI-3 is a 60-item modification of the NEO-FFI-R (Costa & McCrae, 2008; McCrae & Costa, 2004. Five personality trait domains are measures using the NEO-FFI-3, however only the 12-item extraversion measure was implemented in this study. Answers are presented on a 5-point scale from strongly agree to strongly disagree. In the present study, the Cronbach alpha coefficient was good (0.76).

## Results

### Demographic Information

The geographical region of each participant at the time of participation is displayed in Table 1.

**Table 1:** ASD sample compared to TD sample on geographical region at time of participation

	ASD *n* = 230	TD *n* = 272
Country Region	% (*N*)	% (*N*)
Europe	60.4 (n = 139)	81.6 (n = 222)
North America	30.4 (n = 70)	14.3 (n = 39)
South America	0.9 (n = 2)	1.1 (n = 3)
Oceania	6.1 (n = 14)	0.7 (n = 2)
Africa	0.4 (n = 1)	0.0 (n = 0)
Asia	1.3 (n = 3)	0.7 (n = 2)
Other	0.4 (n = 1)	1.5 (n = 4)

### Data Analyses

To investigate if GD symptoms were higher in individuals with ASD, a one-way ANOVA was conducted. The GD variable was skewed, and a logarithmic transformation was performed on the variable prior to the analysis. Following this, a hierarchical regression was conducted to examine the effect of social functioning, peer attachment, extraversion and emotional regulation on GD. To explore whether gelotophobia and GD are related, Pearson’s correlation coefficient was calculated.

### Inferential Statistics

#### ANOVA Analysis

**Fig. 1:**
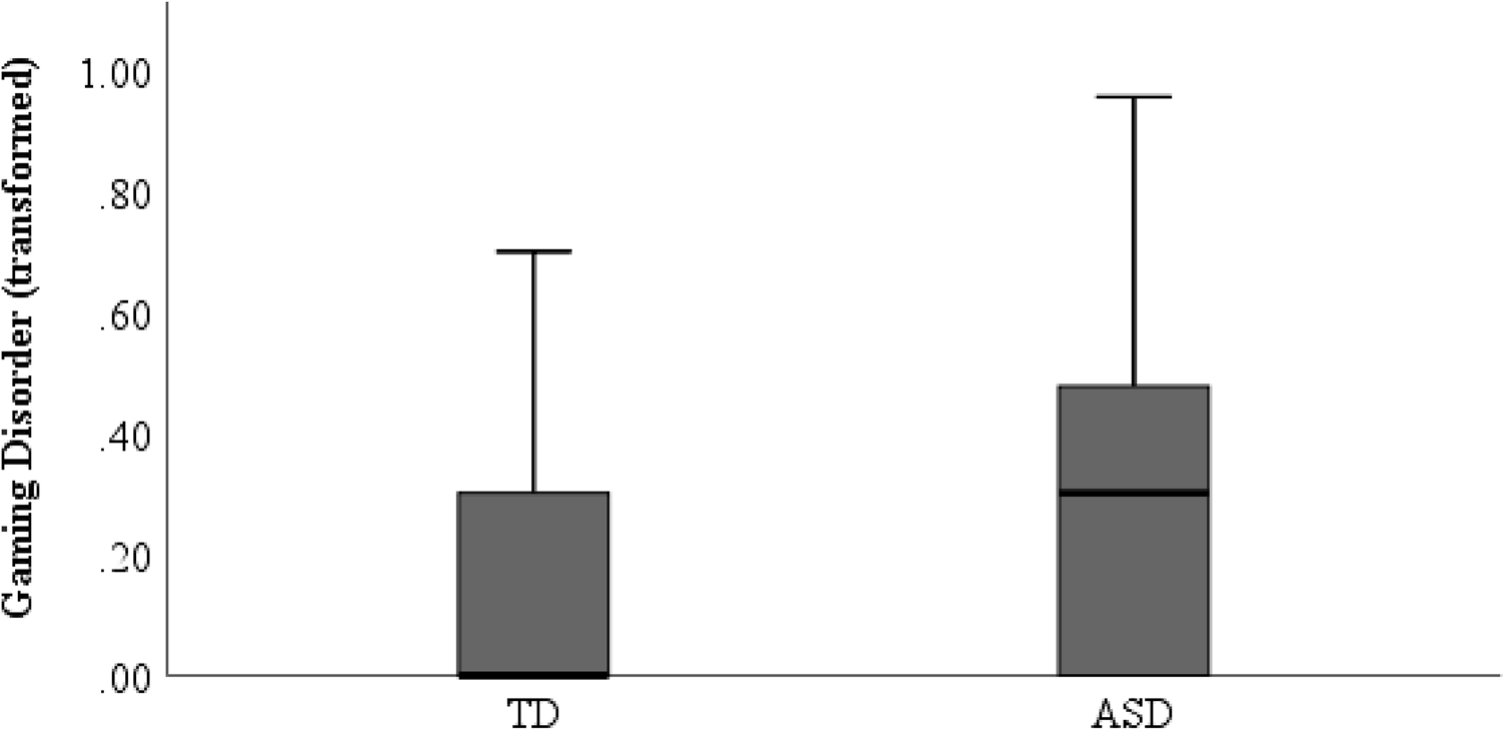
Boxplot comparison of gaming disorder between ASD and TD group

It was found that 9.1% of the ASD group and 2.9% of the TD sample reported symptoms over the cut-off for gaming disorder. The one-way ANOVA found a significant difference between groups in the number of gaming disorder symptoms reported (*F*_(1, 501)_ = 21.42, *p* < 0.001). Participants in the ASD group (*M* = 0.28, *SD* = 0.29) demonstrated higher rates of gaming disorder symptoms than TD participants (*M* = 0.17, *SD* = 0.25) when examining the transformed variable (see Fig. 1). Levene’s test for homogeneity of variance was not significant, based on the mean for GD (*F*_(1, 500)_ = 3.86, *p* = 0.05), ensuring homogeneity of variance.

#### Regression Analysis

To investigate the predictors of GD, a hierarchical regression was conducted for each criterion variable. Extraversion, peer attachment, emotional regulation, and social functioning were entered into each block. Multicollinearity was not present in the data. Pearson’s correlation statistics for predictor variables were less than 0.9. The variance inflation factor (VIF) scores were less than 10 and tolerance scores were greater than 0.1.

Multiple regression analysis showed that the overall model was significant, accounting for 12% of the variance in GD (*F*_(7,478)_ = 25.65, *p* < 0.001). Extraversion in step one was significant, accounting for 6% of the variance in GD (*F*_(1,484)_ = 29.28, *p* < 0.001). Step two, peer attachment also contributed significantly to the model explaining 5% of the variance (*F*_(3,481)_ = 10.04, *p* < 0.001). Step four, emotional regulation accounted for 1% of the variance (*F*_(2,479)_ = 3.45, *p* = 0.033). The final step, social functioning also explained 1% of the variance (*F*_(1,478)_ = 4.42, *p* = 0.036). Extraversion, alienation, cognitive reappraisal, and social functioning were the only significant contributors to the variances explained (see Table 2).

**Table 2:** Summary of hierarchical regression analysis for predictors of gaming disorder

Step	Variable	*β*	Δ*R*^2^	Adjusted Δ*R*^2^	*F* change
1	Extraversion	− .15**	.06	.06	29.28***
2	Trust	− .16	.06	.05	10.04***
	Communication	.01			
	Alienation	.20***			
3	Cognitive reappraisal	− .14**	.01	.01	3.45*
	Expressive suppression	.01			
4	Social functioning	− .10*	.01	.01	4.42*

*Total R*^2^ .13, *adjusted R*^2^ .12

*p < .05

**p < .01

***p < .001

#### Correlation Analysis

**Fig. 2:**
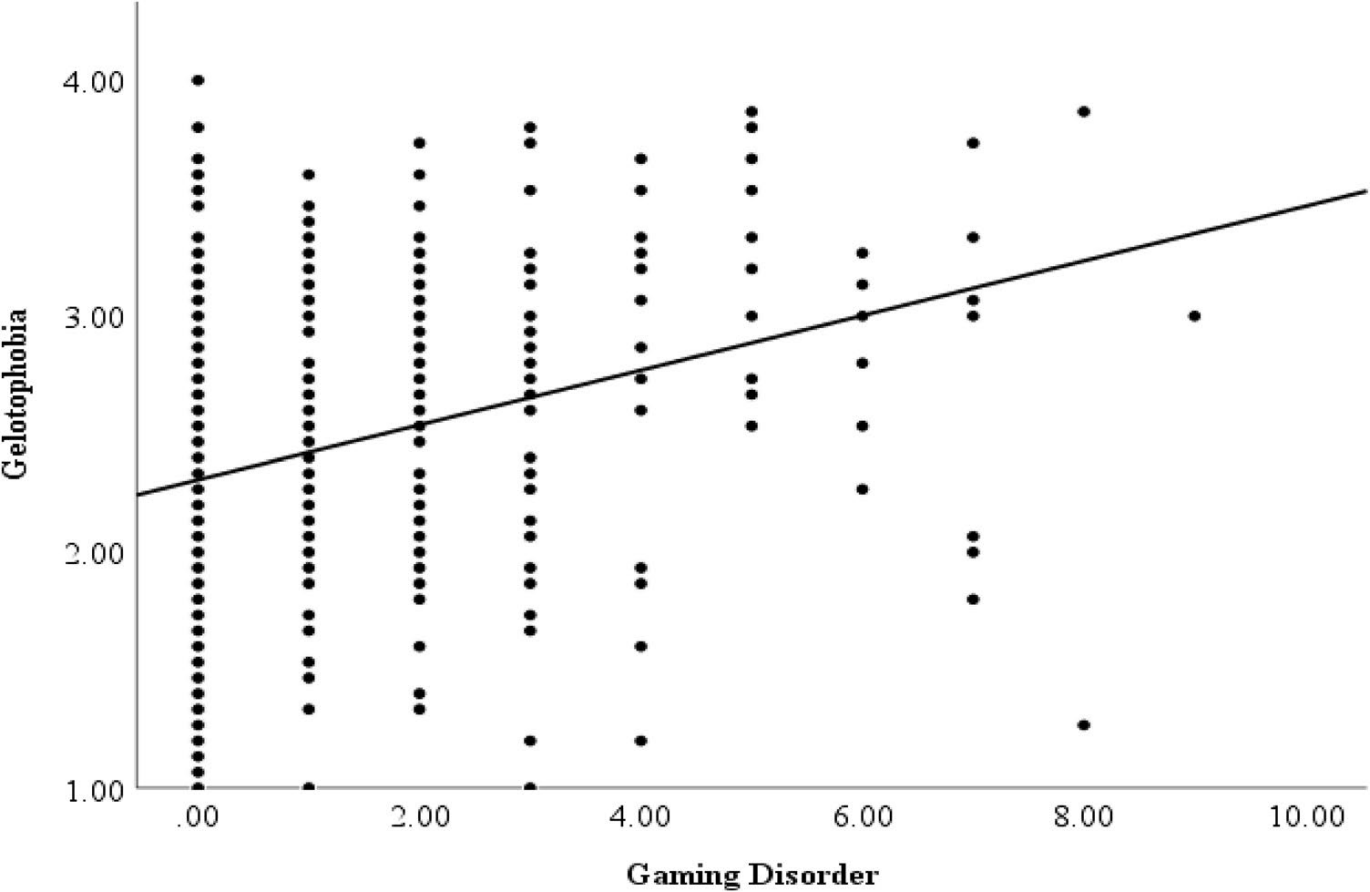
Correlation between gaming disorder and gelotophobia

In relation to our third research question, gelotophobia and GD were significantly correlated (*r* = 0.29, *p* < 0.00). This correlation is displayed in Fig. 2.

## Discussion

The present study investigated the link between ASD and GD, the predictors of GD and the relationship between gelotophobia and GD in adults with and without ASD. As hypothesised, gaming disorder symptoms were significantly higher in participants with ASD compared with the control group with 9.1% of the ASD group and 2.9% of the TD group classified as having GD. No research to date has reported the prevalence of GD in an ASD sample, but the prevalence in the TD group is in line with a previous study which found just below 2% of adults were classified as gaming addicts using the same scale and similar recruitment procedures to our study (Laconi et al., 2017). Our results corroborate findings by Engelhardt et al. (2017) who reported adults with ASD were significantly more likely to develop GD. However, our findings showed a small effect size, whereas Engelhardt et al. (2017) and all previous studies on adolescents reported medium and large effect sizes (MacMullin et al., 2016; Mazurek & Engelhardt, 2013; Mazurek & Wenstrup, 2013; Paulus et al., 2019).

One strength of our study was that we included more female participants in comparison to male participants, whereas previous research has been predominantly male-focused. This may have affected the power of our findings as females have demonstrated less GD symptomology across the literature (Fam, 2018). Furthermore, this is the first study to our knowledge that has inspected GD in an ASD sample which includes participants over 25 years old. Younger individuals may be prone to game more excessively (Mihara & Higuchi, 2017) and hence our inclusion of older participants may be a reason why we found only a small effect size.

Social functioning, extraversion, emotional regulation, and peer attachment each predicted GD. Only the subscale cognitive reappraisal in the emotional regulation scale and alienation in the peer attachment scale were significant in the prediction model. Extraversion negatively predicted GD, indicating that the more introverted a person is, the more likely they may be to develop GD. Similarly, alienation positively predicted GD, which would suggest being alienated from friends and peers could increase the likelihood of a person developing GD as previously found in TD samples (Tonioni et al., 2014).

Lessened cognitive reappraisal abilities predicted GD in this study, supporting previous findings in TD samples (Yen et al., 2018). This suggests that individuals who are less able to alter their emotions by changing their thoughts may be more at risk of developing GD. Better social functioning predicted higher GD scores, which is contradictory to previous research which found lower levels of social functioning were related to higher GD scores (Mihara & Higuchi, 2017). However, social functioning was a weak predictor only accounting for a small amount of variance in GD scores which may explain the discrepancy. Alternatively, a specific social functioning measure has not been used in previous research, only subcomponents of social functioning have been examined, which may explain the difference. The total regression model only explained a small amount of variance indicating there are likely other variables affecting GD symptomatology.

Our study included a novel finding that GD and gelotophobia were related to one another with a small effect size. The two variables had similar relationships with extraversion, emotional regulation and peer attachment in this study. Social anxiety, deficits in understanding emotions, low self-esteem and insecure attachment styles have been associated with higher levels of both GD and gelotophobia, which further suggests they may be related. However, this is the first study to investigate GD and gelotophobia, hence further research is needed to support the finding that they are associated.

Some limitations to this study include the use of self-report measures, convenience sampling, the inclusion of more female participants in comparison to male participants, and the use of a cross-sectional design. Self-report measures can be unreliable and are prone to the exaggeration of conditions such as GD (Maraz et al., 2015). ASD diagnoses were self-reported and were not verified, however those who indicated they were self-diagnosed were excluded. It is also imperative to note that while we included a prevalence rate of GD, this is strictly for descriptive purposes and is not diagnostic in nature. Convenience sampling is a second limitation as gamers may self-select into the study. However, the study aimed to include non-gamers and it was made apparent to participants that they did not need to game to take part. More females were included in comparison to males, leading to a gender imbalance which means our findings may not extend to a general demographic. However, this the frequency of female participants could also be considered a strength of the study. Lastly, as this study implemented a cross-sectional design, no causation can be inferred.

Numerous scales and measures have been used to evaluate GD in individuals with ASD, impacting the comparability of studies. A screening tool for GD based on ICD-11 criteria is being created by an international working group from the WHO at present (Carragher et al., 2019). Future research would benefit from the implementation of this scale across all studies to increase the generalisability and comparability of studies. Research which involves clinical interviews to validate an autism diagnosis, could also extend the interview to clinically diagnose GD according to the criteria set out in the ICD-11, or to evaluate gelotophobia through an interview format.

Age and gender may be influencing the strength of the relationship between ASD and GD. Future research could aim to discern if older individuals with ASD and females with ASD may be less likely to become addicted to gaming. Gelotophobia and GD were related in this study and this relationship should be investigated in future. Gelotophobia may increase the likelihood of developing GD, or GD may increase the likelihood of developing gelotophobia.

This novel study was the first to investigate ASD and GD which included participants over 25 years old and included a range of predictor variables. Our findings indicated that individuals with ASD may indeed be more likely to develop GD. Extraversion, alienation and cognitive reappraisal emerged as the biggest predictors of GD. Future research should investigate clinically diagnosed GD and ASD, gender and age differences in GD, and further establish prevalence rates and risk factors of GD in both ASD and TD samples. The unique finding that gelotophobia and GD may be related to one another needs to be further examined in future research studies.
